# CDK4/6 inhibitors improve the anti-tumor efficacy of lenvatinib in hepatocarcinoma cells

**DOI:** 10.3389/fonc.2022.942341

**Published:** 2022-07-22

**Authors:** Graziana Digiacomo, Claudia Fumarola, Silvia La Monica, Mara Bonelli, Andrea Cavazzoni, Maricla Galetti, Rita Terenziani, Kamal Eltayeb, Francesco Volta, Silvia Zoppi, Patrizia Bertolini, Gabriele Missale, Roberta Alfieri, Pier Giorgio Petronini

**Affiliations:** ^1^ Department of Medicine and Surgery, University of Parma, Parma, Italy; ^2^ Department of Occupational and Environmental Medicine, Epidemiology and Hygiene, INAIL - Italian Workers’ Compensation Authority, Rome, Italy; ^3^ Paediatric Hematology Oncology Unit, University Hospital of Parma, Parma, Italy; ^4^ Unit of Infectious Diseases and Hepatology, University Hospital of Parma, Parma, Italy

**Keywords:** hepatocarcinoma (HCC), CDK4/6 inhibition, abemaciclib, lenvatinib, senescence

## Abstract

Hepatocellular carcinoma (HCC) is the most frequent primary liver cancer with a poor prognosis and limited treatment options. Considering that alterations of the CDK4/6-cyclin D-Rb pathway occur frequently in HCC, we tested the efficacy of two CDK4/6 inhibitors, abemaciclib and ribociclib, in combination with lenvatinib, a multi-kinase inhibitor approved as first-line therapy for advanced HCC, in a panel of HCC Rb-expressing cell lines. The simultaneous drug combinations showed a superior anti-proliferative activity as compared with single agents or sequential schedules of treatment, either in short or in long-term experiments. In addition, the simultaneous combination of abemaciclib with lenvatinib reduced 3D cell growth, and impaired colony formation and cell migration. Mechanistically, these growth-inhibitory effects were associated with a stronger down-regulation of c-myc protein expression. Depending on the HCC cell model, reduced activation of MAPK, mTORC1/p70S6K or src/FAK signaling was also observed. Abemaciclib combined with lenvatinib arrested the cells in the G1 cell cycle phase, induced p21 accumulation, and promoted a stronger increase of cellular senescence, associated with elevation of β-galactosidase activity and accumulation of ROS, as compared with single treatments. After drug withdrawal, the capacity of forming colonies was significantly impaired, suggesting that the anti-tumor efficacy of abemaciclib and lenvatinib combination was persistent.

Our pre-clinical results demonstrate the effectiveness of the simultaneous combination of CDK4/6 inhibitors with lenvatinib in HCC cell models, suggesting that this combination may be worthy of further investigation as a therapeutic approach for the treatment of advanced HCC.

## Introduction

Hepatocellular carcinoma (HCC) is the sixth most common malignant tumor and the fourth leading cause of cancer-related death (1). HCC is a highly heterogenic disease, and a significant proportion of HCC patients show intermediate/advanced disease at the time of clinical diagnosis, thus reducing the opportunity for a radical cure (2). In these patients, systemic therapy remains the only available therapeutic option.

Sorafenib has been the first drug approved as standard first-line treatment for advanced HCC; however, during the last 4 years, other treatments reached the Food and Drug Administration (FDA) approval, including new molecular targeted agents and immune checkpoint inhibitors (ICIs) (3–5)

The IMbrave150 trial (6) demonstrated that the combination of atezolizumab, an ICI directed against PD-L1, with the anti-angiogenic drug bevacizumab improved the overall survival of HCC patients as compared to sorafenib. Atezolizumab plus bevacizumab has become the standard of care for the treatment of patients with unresectable locally advanced or metastatic HCC (5) However, 20% of patients do not respond to this therapy and the median progression-free survival is only 6.8 months. In addition, there is a portion of patients who cannot be treated with atezolizumab/bevacizumab, for whom the therapy options available remain sorafenib or lenvatinib. Lenvatinib is a multi-kinase inhibitor approved in 2018 as first-line treatment for advanced HCC (7), exerting its inhibitory activity against vascular endothelial growth factor receptors (VEGFR1-3), fibroblast growth factor receptors (FGFR1-4), platelet-derived growth factor receptor alpha (PDGFR-α), c-KIT, and RET (8). Targeting the FGF receptors and inhibiting the FGF signaling pathway distinguishes lenvatinib from sorafenib.

Preclinical studies demonstrated that the combination of lenvatinib with pembrolizumab is effective in HCC, resulting in increased antitumor activity as compared to monotherapy in a mouse model of HCC (9). Interestingly, such combination showed promising antitumor activity with a tolerable safety profile also in patients with unresectable HCC (10).

In patients who failed first-line therapy, the treatment choices in second-line include the multi-tyrosin kinases inhibitors (TKIs) regorafenib and cabozantinib, and the monoclonal anti-VEGF2 antibody ramucirumab (11).

Cell cycle dysregulation is a hallmark of cancer and alterations in genes regulating cell proliferation have been frequently reported in HCC cells. Among them, alterations of the cyclin D-CDK4/6-Rb pathways frequently occur in HCC: CDK4 overexpression has been described in more than 70% of cases (12), whereas the inactivation of *CDKN2A/ARF* gene, encoding for the cell cycle inhibitor p16^INK4a^, has been found in over 50% of patients (13, 14). In addition, the oncosuppressor Rb gene has been found inactivated in around 20% of patients (13, 15).

To date, the CDK4/6 inhibitors palbociclib (PD-0332991), ribociclib (LEE011), and abemaciclib (LY835219) are approved for the treatment of Estrogen Receptor positive advanced or metastatic breast cancer in association with endocrine therapy (16). Interestingly, a number of preclinical studies have demonstrated their efficacy also in Rb-expressing HCC cells (17). Accordingly, our recent findings demonstrated that HCC cell lines harboring *CDKN2A/ARF* gene loss and expressing a functional Rb protein were sensitive to palbociclib, and proved the efficacy of CDK4/6 inhibition in combination with regorafenib, thus suggesting a novel approach for the treatment of HCC patients (18).

Based on these positive findings, we investigated the anti-tumor activity of the combination of CDK4/6 inhibitors with lenvatinib in a panel of HCC cell lines. We focused in particular on abemaciclib, because of its highest potency; in addition, abemaciclib is more potent against CDK4 than CDK6, which is involved in the differentiation of hematologic precursor cells, implying a reduced hematological toxicity in the clinical setting (19). Our results demonstrated that the simultaneous drug combination inhibited cell proliferation in either two or three-dimensional (2D, 3D) cell models, impaired colony formation and cell migration, and induced cellular senescence more strongly than single treatments. Interestingly, the growth-inhibitory effect of the drug combination was maintained even after drug removal, providing a pre-clinical rationale for the combination of abemaciclib with lenvatinib as a therapeutic strategy for advanced HCC.

## Material and methods

### Cell culture

Human HCC cell lines (HUH7, SNU398, HepG2) were obtained from the American Type Culture Collection (ATCC, Manassas, VA); ATCC authenticates the phenotypes of these cell lines on a regular basis. The cells were cultured in Dulbecco’s Modified Eagle Medium (DMEM) supplemented with 2 mM glutamine, 10% Fetal Bovine serum (FBS), and 100 U/ml penicillin,100 μg/ml streptomycin, and incubated at 37°C in a humidified atmosphere of 5% CO_2_ in air.

### Drug treatments

Abemaciclib (S5716), ribociclib (S7740), and lenvatinib (S1164) were purchased from Selleckchem (Houston, TX). All drugs were dissolved in DMSO. DMSO concentration never exceeded 0.1% (v/v); equal amounts of the solvent were added to control cells.

### Western blotting

Western blot analysis was performed as previously described (20). Antibodies against p-Rb^Ser780^, Rb, cyclin D1, CDK6, p-ERK1/2^Thr202/Tyr204^, ERK1/2, p-AKT^Ser473^, AKT, p-p70S6K^Thr389^, p70S6K, p-FAK^Tyr925^, FAK, p-SrcTyr^416^, Src, PDGFRα, FGFR1, FGFR2, cKIT, *c*-Myc, p21, p-MDM2 and MDM2 were from Cell Signaling Technology, Incorporated (Danvers, MA); anti-p-CDK6^Tyr24^, FGFR4 were from Santa Cruz Biotechnology, Incorporated (Dallas, TX). The antibody against CDKN2A/p16^INK4a^ was from Abcam (Cambridge, UK). Anti-β-actin (clone B11V08) was from BioVision (Milpitas, CA). Horseradish peroxidase-conjugated secondary antibodies and the chemiluminescence system were from Millipore (Millipore, MA). Reagents for electrophoresis and blotting analysis were from BIO-RAD Laboratories (Hercules, CA). The chemiluminescent signal was acquired by C-DiGit R Blot Scanner and the bands were quantified by Image Studio™Software, LI-COR Biotechnology (Lincoln, NE).

### Drug combination studies

The nature of the interaction between CDK4/6 inhibitors and lenvatinib was calculated by combination index (CI) determination. CIs were calculated with Calcusyn software (Biosoft), which is based on the method of Chou and Talalay. In this method, a CI < 0.8 is considered as synergistic, 0.8 < CI < 1.2 additive and CI > 1.2 antagonistic (21).

### Colony and clonogenic assay

Cells were seeded in 12-well culture plates at a low density and were incubated at 37°C in 5% CO_2_ incubator. After 6 days of treatment, cells were fixed with ice-cold methanol, stained with 0.1% crystal violet (Sigma Aldrich). The unbound dye was removed by washing with water. The bound crystal violet was solubilized with 0.2% TritonX-100 in PBS and the absorbance of the solution was measured at a wavelength of 570 nm (22). In the experiments of clonogenic assay, after 6 days of treatment, viable cells were harvested, reseeded in 6-well culture plates at a density of 400 cells per well, and cultured for additional 15 days in the absence of drugs. After the recovery time, colonies were fixed with methanol, stained with crystal violet and counted. Data were given as colony number (23).

### Analysis of spheroid cell growth

Spheroids from HCC cells were generated using LIPIDURE^®^-COAT PLATE A-U96 (NOF Corporation, Japan) according to the manufacturer’s instructions. Briefly, 3 days after seeding, the spheroids were formed and treated with drugs or vehicles for 6 or 12 days. The effects of the drugs were evaluated in terms of volume changes using the Nikon Eclipse E400 Microscope with digital Net camera. The volume of spheroids [D=(Dmax+Dmin)/2; V=4/3π(D/2)3] was measured using SpheroidSizer, a MATLAB-based and open-source software application (24).

### Migration

The migration assay was carried out using Transwell chambers with 6.5-mm diameter polycarbonate filters (8μm pore size, BD Biosciences, Erembodegem, Belgium) as previously described (25).

### β-Galactosidase staining

The evaluation of Senescence Associated β-Galactosidase (SA-β-Gal) expression was performed using the Senescence β-Galactosidase Staining kit (Cell Signaling Technology Inc.). Briefly, cells were seeded in 6-well plates in complete medium for 24 h and then treated with drugs or vehicles. At the end of the treatments, a fixative solution was added for 15 min at room temperature. The plates were washed twice with PBS and then the β-Gal staining solution was added. After overnight incubation at 37°C without CO_2_, the number of SA-β-Gal positive cells (blue stained) was evaluated by cell counting in ten randomly chosen microscope fields (100× magnification) (26).

### Reactive oxygen species detection

Intracellular ROS production was assessed by oxidation of the cell-permeable fluorescent probes 5-(and-6)-chloromethyl-20,70-dichlorodihydrofluorescein diacetate, acetyl ester (CM-H2DCFDA, Molecular Probes^®^). At the end of the drug treatments, the cells were harvested, centrifuged, resuspended in PBS containing the probe at 1 µM, and incubated in the dark at 37°C. After 30 minutes, the cells were washed, resuspended in PBS, and analyzed on a Beckman-Coulter Cytoflex flow cytometer.

### Statistical analysis

Statistical analyses were carried out using Graph-Pad Prism version 6.0 software (GraphPad Software, San Diego, CA). Statistical significance of differences among data was estimated by analysis of variance (ANOVA) followed by Tukey’s post-test. p values are indicated where appropriate in the figures and in their legends. p values less than 0.05 were considered significant.

## Results

### Effects of abemaciclib alone or combined with lenvatinib in HCC cell lines

**Figure 1:**
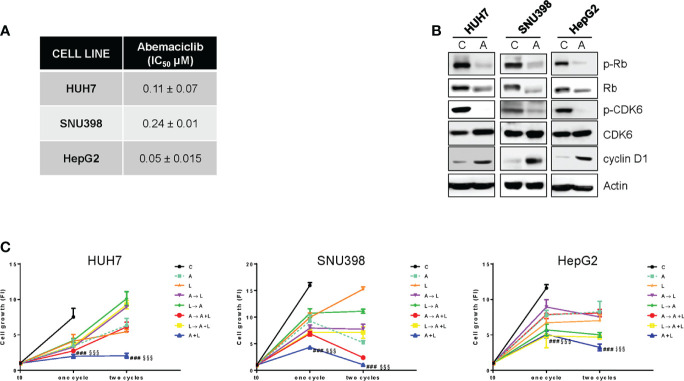
The simultaneous combined treatment of abemaciclib and lenvatinib inhibits cell proliferation more strongly than single agents and sequential schedules in HCC cells. **(A)** After 24h from seeding, HUH7, SNU398, and HepG2 cells were treated with increasing concentrations of abemaciclib (A) for 6 days. Cell proliferation was evaluated by CV assay and the IC_50_ values were calculated using GraphPad Prism 6.00 software. **(B)** HCC cells were untreated (Control, C) or treated with 1 μM A for 24h. The cells were lysed and the expression of the indicated proteins was evaluated by Western blot analysis. **(C)** HUH7, SNU398, and HepG2 cells were treated with A or L at their corresponding IC50 values for 6 days (one cycle), or 12 days (two cycles), alone or with different combinations of the two drugs: A for 72h followed by L for 72h (A→L), L for 72h followed by A for 72h (L→A), A for 72h followed by A+L for 72h (A→A+L), L for 72h followed by A+L for 72h (L→A+L), or a simultaneous combination for 6 days (A+L) for each cycle. The growth medium with drugs was refreshed every 3 days. Cell proliferation was evaluated by CV assay. Data are expressed as Fold Increase (FI). The FI index was calculated as the ratio between cell proliferation after 6 or 12 days and cell proliferation at T_0_ (T_0 =_ 24h after seeding). ^###^p<0.001 vs A; ^§§§^p<0.001, vs L. Data in A are mean values ± SD of three independent experiments. Data in B-C are representative of two independent experiments.

Firstly, we demonstrated that abemaciclib was effective at inhibiting cell proliferation in a panel of HCC cell lines (**Figure 1A**). As previously demonstrated for HUH7 and HepG2 cells (18), SNU398 cells also expressed a functional Rb protein and were negative for p16^INK4a^ expression (**Figure S1A**), molecular features that have been previously correlated with sensitivity to CDK4/6 inhibition (20, 27). In these cells, abemaciclib decreased CDK6 phosphorylation and downregulated both p-Rb and Rb levels (**Figure 1B**), in accordance with data from literature showing that CDK4/6 inhibitors reduce Rb phosphorylation and concomitantly decrease the expression of total Rb protein in multiple cancer models (28). In addition, abemaciclib increased cyclin D1 levels (**Figure 1B**), an effect that we had previously observed with palbociclib treatment (18).

Then, we investigated whether abemaciclib treatment could potentiate the anti-tumor activity of lenvatinib. To this end, we firstly analyzed the effects of lenvatinib alone in HCC cells and demonstrated that it inhibited cell proliferation, with a stronger efficacy observed in HUH7 cells compared with HepG2 and SNU398 cells (**Figure S1B**), confirming results from previous studies (29). All three cell models expressed one or more TK receptors targeted by lenvatinib (**Figure S1C**): in particular, FGFR1, FGFR2, and FGFR4 were expressed in all cell lines, although to a different extent, c-KIT was absent in SNU398, while PDGFR-α was detected only in HUH7 cells, presumably contributing to their greater sensitivity to lenvatinib. These data confirm that lenvatinib, in addition to its anti-angiogenic activity, exerts direct anti-tumor effects in cells expressing its targets.

The combination of abemaciclib with lenvatinib was evaluated by comparing a simultaneous treatment with sequential schedules (abemaciclib followed by lenvatinib; lenvatinib followed by abemaciclib; abemaciclib or lenvatinib followed by the simultaneous drug combination). In all cell lines analyzed, the simultaneous combination induced a greater inhibition of cell proliferation compared with single agents, and was more efficacious than the sequential schedules, after one or two cycles of treatment (**Figure 1C**). Actually, some of the sequential schedules were even less effective than single-agent treatments. Based on these findings, we chose the simultaneous combined treatment with abemaciclib and lenvatinib for the subsequent experiments.

**Figure 2:**
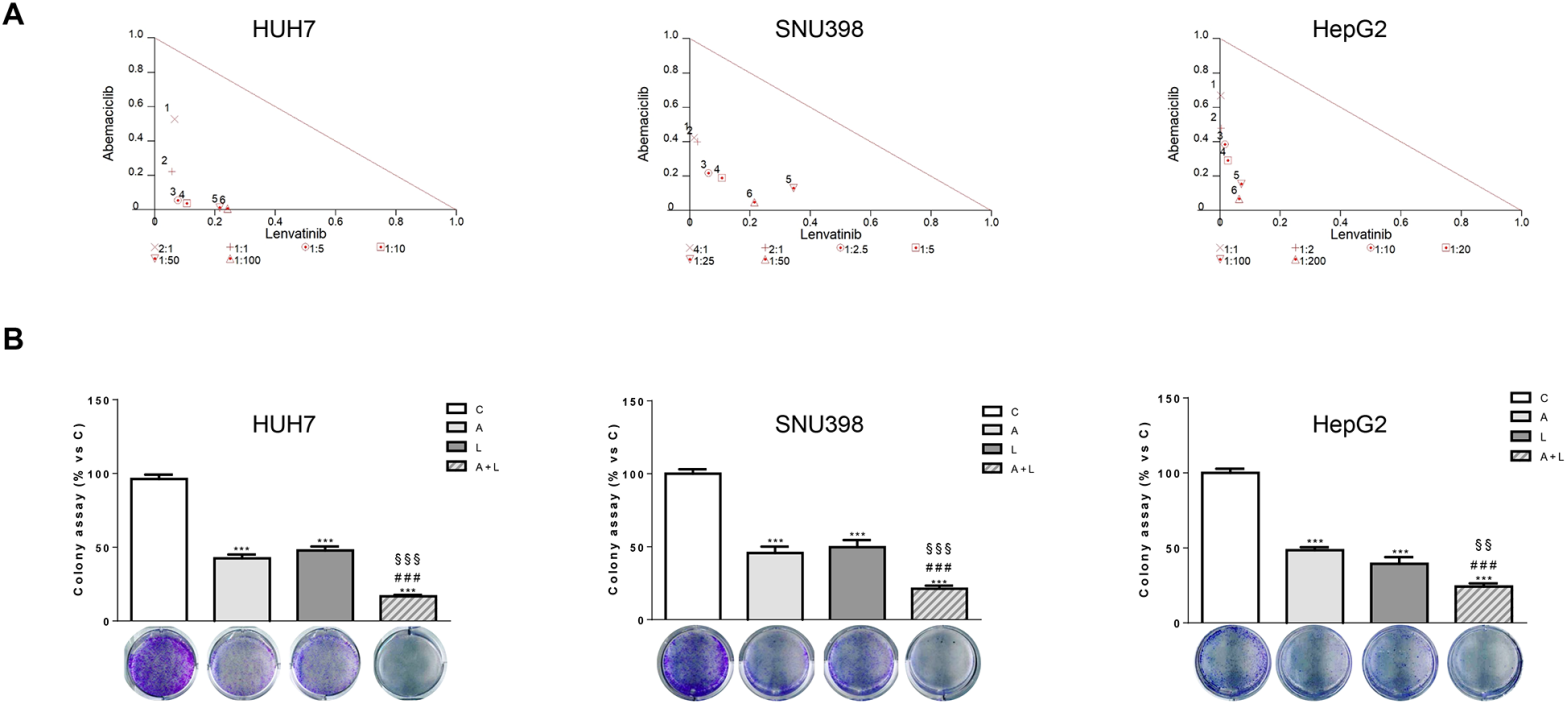
Abemaciclib and lenvatinib combination exerts additive anti-proliferative effects in HCC cells and inhibits colony formation more strongly than single agents. **(A)** Cells were treated with A, L or the combination. The growth medium with drugs was refreshed every 3 days. After 6 days, cell proliferation was assessed by CV assay. Combination indexes were calculated with Calcusyn software. **(B)** HUH7, SNU398, and HepG2 cells were treated with A or L at their corresponding IC_50_ values alone or in combination. After 6 days, colony formation was assessed by CV assay. Representative images of crystal violet staining of colonies are shown. ***p<0.001 vs C, ^###^p<0.001 vs A; ^§§^p< 0.01, ^§§§^p<0.001 vs L. Data in A are representative of three independent experiments. Data in B are mean values ± SD of three independent experiments.

To identify the type of interaction between abemaciclib and lenvatinib, we calculated the combination index (CI). As shown in **Figure 2A**, we demonstrated that this combination resulted in synergistic anti-proliferative effects in HUH7, SNU398, and HepG2 cells.

The superior efficacy of abemaciclib combined with lenvatinib was also demonstrated by a cell colony formation assay, showing that the combination reduced the colony formation more strongly than single-agent treatments in all three cell models analyzed (**Figure 2B**).

### Effects of ribociclib alone or combined with lenvatinib in HCC cell lines

**Figure 3:**
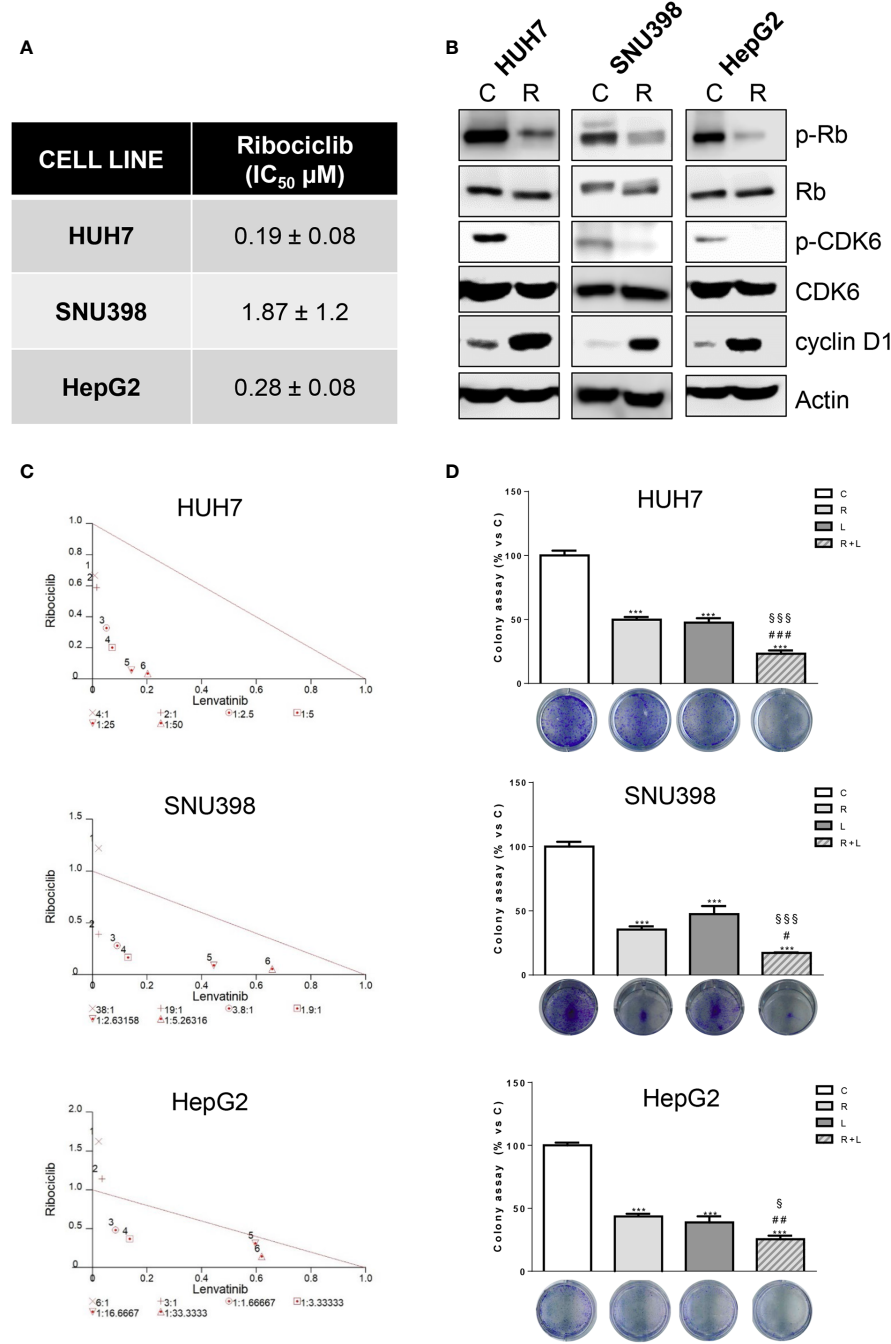
Ribociclib and lenvatinib combination exerts additive anti-proliferative effects in HCC cells and inhibits colony formation more strongly than single agents. **(A)** After 24h from seeding, HUH7, SNU398, and HepG2 cells were treated with increasing concentrations of ribociclib (R) for 6 days. Cells proliferation was evaluated by CV assay and the IC_50_ values were calculated using GraphPad Prism 6.00 software. **(B)** HCC cells were untreated (C) or treated with 1 μM R for 24h. The cells were lysed and the expression of the indicated proteins was evaluated by Western blot analysis. **(C)** Cells were treated with R, L or the combination. The growth medium with drugs was refreshed every 3 days. After 6 days, cell proliferation was assessed by CV assay. Combination indexes were calculated with Calcusyn software. **(D)** HUH7, SNU398, and HepG2 cells were treated with R or L at their corresponding IC_50_ values alone or in combination. After 6 days, colony formation was assessed by CV assay. Representative images of crystal violet staining of colonies are shown. ***p<0.001 vs C; ^#^p<0.05, ^##^p<0.01 ^###^p<0.001 vs R; ^§^p< 0.05, ^§§§^p<0.001 vs L. Data in A are mean values ± SD of three independent experiments. Data in B-C are representative of two independent experiments. Data in D are mean values ± SD of two independent experiments.

Then, we evaluated whether the same increased anti-tumor activity demonstrated by abemaciclib in combination with lenvatinib could be achieved by inhibiting CDK4/6 with ribociclib. Ribociclib inhibited cell proliferation in the HCC cell models, downregulating p-CDK6 and p-Rb, while cyclin D1 levels were increased as shown for abemaciclib (**Figures 3A, B**).

The simultaneous combination with lenvatinib produced synergistic anti-proliferative effects, (**Figure 3C**) and was more effective than single treatments in reducing the colony formation (**Figure 3D**). It is worth noting that the IC_50_ value for ribociclib was higher than that determined for abemaciclib in all three cell models; actually abemaciclib shows a higher potency in comparison to the other CDK4/6 inhibitors (19). Therefore, we decided to continue using abemaciclib to investigate the effects of CDK4/6 inhibition in combination with lenvatinib.

### The combination of abemaciclib with lenvatinib inhibits spheroid cell growth, impairs cell migration, and induces cell cycle arrest and senescence

**Figure 4:**
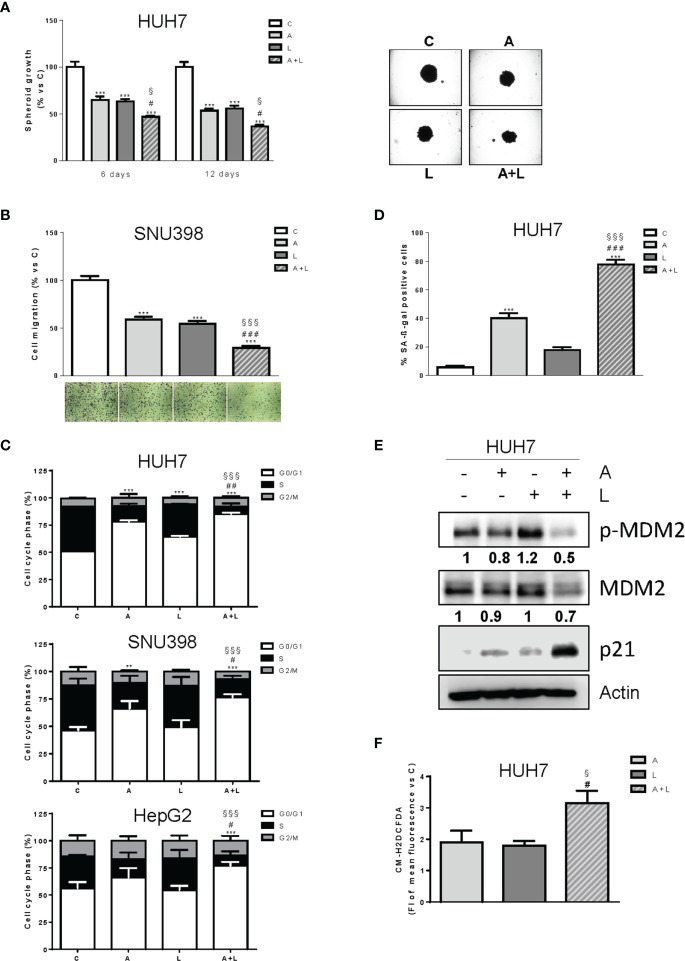
Abemaciclib combined with lenvatinib reduces 3D cell growth, inhibits cell migration, and induces G1 cell cycle arrest associated with senescence. **(A)** The growth of spheroids from HUH7 was analyzed after 6 or 12 days of treatment with 0.1 μM A and 0.5 μM L alone or in combination. The data are expressed as percent of spheroid growth versus control. Representative images of spheroids after 12 days of culture are shown. ***p<0.001 vs C; ^#^p<0.05 vs A; ^§^p<0.05 vs L. **(B)** SNU398 cells were treated with 0.2 μM A or 2 μM L as single treatment or in combination for 20h. Migrated cells were then counted. Data are expressed as percent versus control. Representative fields of migration are shown (magnification of 4×). ***p<0.001 vs C; ^###^p<0.001 vs A; ^§§§^p<0.001 vs L. **(C)** HUH7, SNU398, and HepG2 cells were untreated (C) or treated with 0.1, 0.2 μM and 0.05 μM A and 0.5, 2 or 6.5 μM L for HUH7, SNU398 and HepG2, respectively, as single treatment or in combination for 24h. After 24h the cells were stained with propidium iodide and cell-cycle-phase distribution was determined by flow cytometry. Data are expressed as percentage of cells in each cell-cycle phase. **p<0.01, ***p<0.001 vs C; ^#^p<0.05, ^##^p<0.01 vs A; ^§§§^p<0.001 vs L. **(D)** HUH7 cells were treated with 0.1 μM A and 0.5 L alone or in combination for 6 days. Histograms represent the percentage of senescent cells positive for SA-β-Gal expression. ***p<0.001 vs C; ^###^p<0.001 vs A; ^§§§^p<0.001 vs L. **(E)** HUH7 was treated with 0.1 μM A and 0.5 L alone or in combination for 3 days. The cells were lysed and the expression of the indicated proteins was evaluated by Western blot analysis. **(F)** HUH7 were treated with 0.1 μM A and 0.5 L alone or in combination. After 6 days, the cells were incubated with 1 μM CM-H2DCFDA probe and analyzed by flow cytometry. ^#^p<0.05 vs A; ^§^p<0.05 vs L. Experiments in A-E are representative of two independent experiments. Data in **(B, C)** are mean values ± SD of two independent experiments. Data in D are mean values ± SD of three independent experiments. Data in F are mean values ± SD of three independent experiments.

The greater efficacy of the simultaneous combined treatment with abemaciclib and lenvatinib over individual agents was further confirmed in a 3D system using HUH7 cells (**Figure 4A**). Indeed, both drugs alone inhibited spheroid cell growth but were significantly more effective when used in combination.

Interestingly, abemaciclib combined with lenvatinib significantly impaired the ability of SNU398 cells to migrate (**Figure 4B**).

The simultaneous combination induced cell cycle arrest (**Figure 4C**), with an increased percentage of cells accumulating in the G1 phase in comparison with abemaciclib or lenvatinib alone. No significant induction of cell death was observed (not shown). Interestingly, abemaciclib but not lenvatinib significantly induced senescence, as demonstrated by the induction of β-gal activity in HUH7 cells, and the percentage of senescent cells was further increased after the simultaneous treatment (70% versus 40%) (**Figure 4D**). Mechanistically, the combination induced significant higher levels of p21 protein (**Figure 4E**), which is known to play a key role in the initiation of senescence-mediated growth arrest (30). Since HUH7 cells express a Y220C-mutated form of p53 (31), we investigated the mechanisms responsible for p21 accumulation, analyzing the activation/expression of MDM2, which has been demonstrated to negatively regulate p21, activating its proteasomal-mediated degradation independently of p53 (32). As shown in **Figure 4E**, MDM2 phosphorylation and total protein expression were significantly downregulated by abemaciclib/lenvatinib combination, providing a mechanism for p21 induction. Since a variety of evidence indicates that accumulation of intracellular ROS contributes to senescence (33), we evaluated whether the emergence of the senescent phenotype in abemaciclib/lenvatinib-treated cells was related to increased ROS levels. To this end, we measured ROS production by using the fluorescent probe CM-H2DCFDA and demonstrated that the drug combination effectively promoted a stronger increase in ROS levels compared with single-agent treatments (**Figure 4F**).

### The combination of abemaciclib with lenvatinib has different modulatory effects on intracellular signaling pathways depending on the cell model

**Figure 5:**
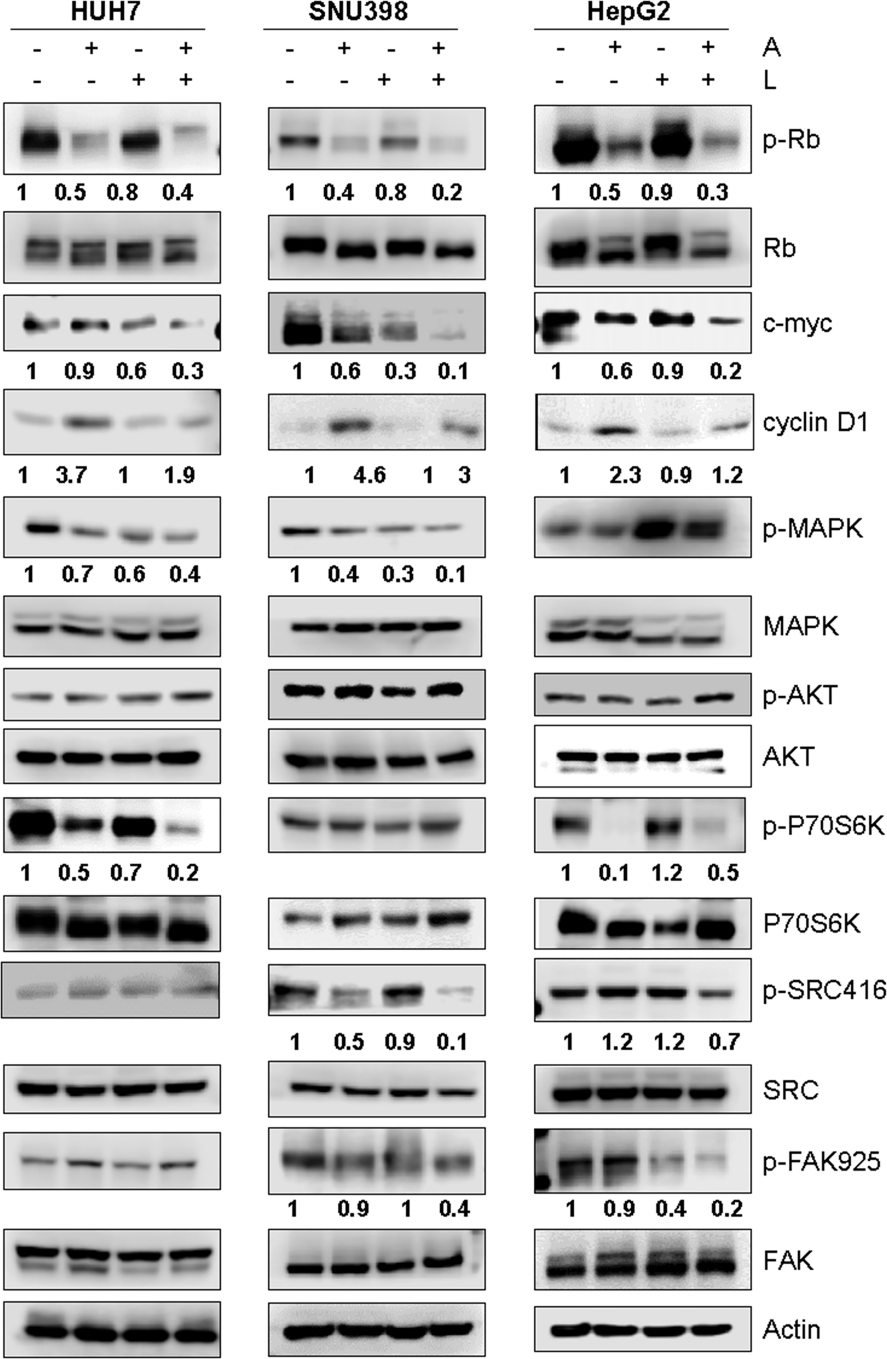
Effects of the simultaneous combination of abemaciclib and lenvatinib on intracellular signaling pathways. Cells were treated with 1 μM A and 0.5, 2, or 6.5 μM L for HUH7, SNU398, and HepG2, respectively, alone or in combination for 24 h. The cells were lysed and the expression of the indicated proteins was evaluated by Western blot analysis. Data are representative of two independent experiments.

Then, we investigated the molecular mechanisms underlying the superior efficacy of the drug combination (**Figure 5**). Rb phosphorylation was reduced not only by abemaciclib but also by lenvatinib, although to a lesser extent, in all three HCC cell lines; interestingly, a further downregulation was induced by the combination, resulting in a stronger inhibition of c-myc expression, whose transcriptional modulation by E2F is well-recognized (34). In addition, the combination significantly reduced the abemaciclib-mediated increase of cyclin D1 expression levels. While the inhibitory effects on the expression of cell cycle regulatory proteins were comparable in the three cell lines, the impact of abemaciclib/lenvatinib combination on the activation status of survival/proliferation intracellular signaling pathways varied significantly depending on the cell model.

In HUH7 cells, the phosphorylation of the mTORC1 substrate p70S6K was reduced by both abemaciclib and lenvatinib, and further downregulated by the combined treatment, even though p-AKT levels were not affected. These inhibitory effects were a consequence of the inhibition of the MAPK pathway, which can modulate the mTORC1/p70S6K signaling independently of AKT (35), as confirmed by using the selective MEK1/2 inhibitor trametinib (**Figure S2A**). The drug combination downregulated the MAPK pathway also in SNU398 cells, although without affecting the mTORC1/p70S6K signaling; p-AKT levels did not change. Accordingly, trametinib did not inhibit p70S6K phosphorylation in these cells (**Figure S2B**), suggesting that the AKT-independent mechanism of mTORC1 modulation by the MAPK pathway is cell type-specific. In HepG2 cells, abemaciclib alone downregulated p-p70S6K levels, supporting the finding that CDK4/6 inhibition reduces mTORC1 activity in some cancer models (36); however, no further decrease was observed with the combination. AKT phosphorylation was unchanged, while p-ERK1/2 levels were increased by lenvatinib alone or in combination. Interestingly, a significant downregulation of src/FAK signaling was promoted by abemaciclib and lenvatinib combination in SNU398 and HepG2 cells. Due to the key role of this pathway in the control of cell migration (37), its inhibition was presumably responsible for the impairment of cell migration observed in SNU398 cells.

### The anti-proliferative effects of abemaciclib with lenvatinib are maintained after drug removal

**Figure 6:**
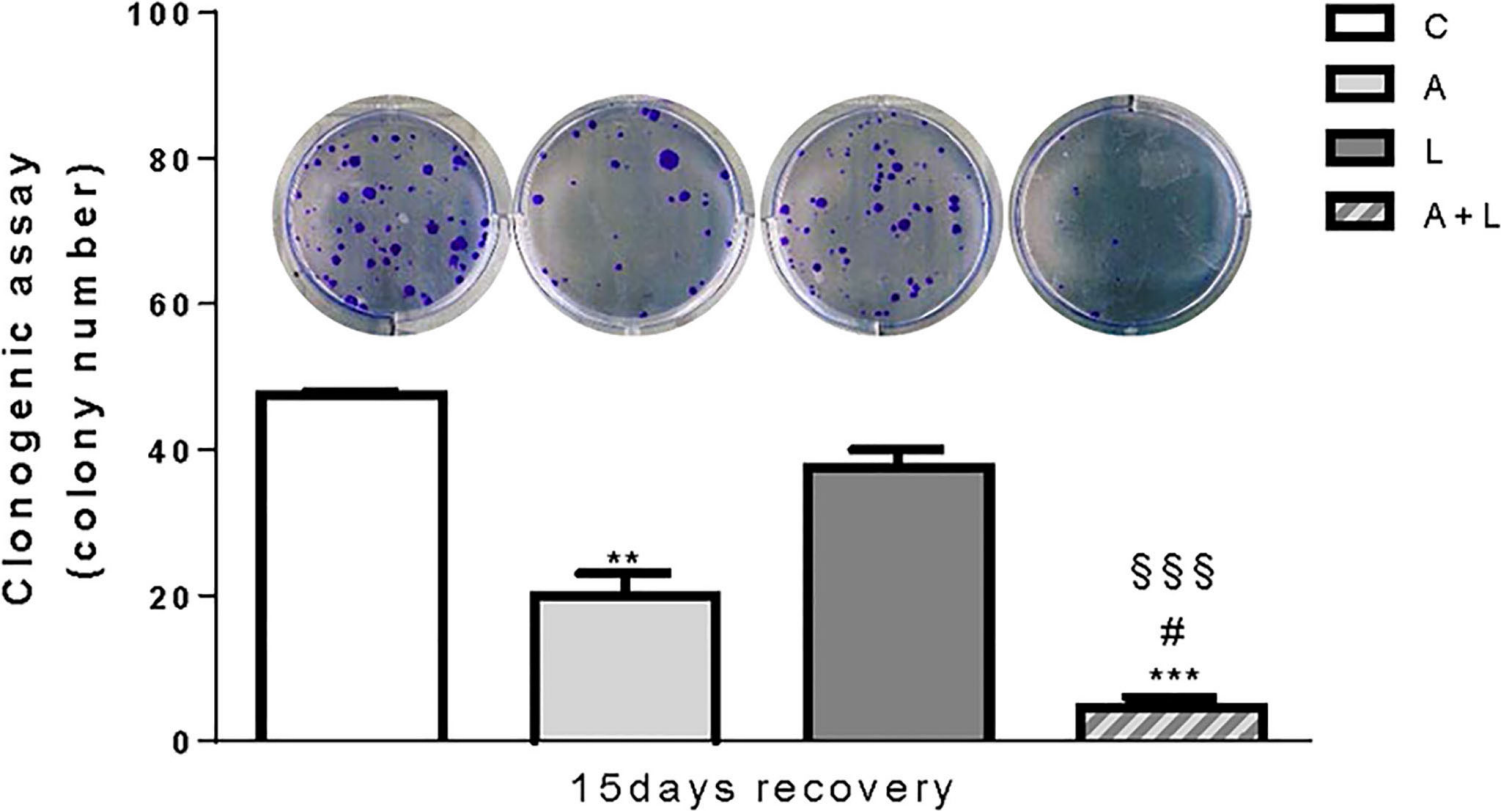
The anti-proliferative effects of abemaciclib and lenvatinib combination persisted after drug withdrawal. HUH7 after 6 days of drug treatments, the cells were harvested, seeded at low density, and cultured for additional 15 days in the absence of the drugs (recovery). Representative images of crystal violet staining of colonies are shown. **p<0.01,***p<0.001 vs C; ^#^p<0.05 vs A; ^§§§^p<0.001 vs L. Data are mean values ± SD of two independent experiments.

To evaluate whether the anti-tumor effects of abemaciclib and lenvatinib combination were persistent, we performed long-term experiments in HUH7 cells. After 6 days of treatment with the drugs alone or combined, the cells were harvested, counted, reseeded at low density, and cultured in drug-free medium for additional 15 days to test their capacity to form colonies. As shown in **Figure 6**, colony formation was significantly impaired in cells previously treated with abemaciclib, whereas the inhibitory effects of lenvatinib were almost completely lost. Most importantly, in the culture of cells previously exposed to the drug combination, very few and small colonies were detected, suggesting that the superior efficacy of the combined treatment is maintained even after drug removal.

## Discussion

Despite the recent advances in the management of advanced HCC, this malignant disease remains difficult to treat and the development of more effective therapies is warranted. In this regard, different strategies are currently under evaluation, including approaches exploring the anti-cancer properties of compounds normally used for other medical purposes (38), or combinatorial approaches aimed at improving the efficacy of the therapies used in the clinical practice (5).

In this study, we provide evidence that the simultaneous combination of the CDK4/6 inhibitors abemaciclib and ribociclib with lenvatinib is effective in Rb-proficient, p16^INK4^ negative HCC cells, producing synergistic anti-proliferative effects that persisted even after drug withdrawal. These findings are in accordance with our previous results showing that palbociclib, another CDK4/6 inhibitor, improved the efficacy of regorafenib in HCC cells (18), suggesting that CDK4/6 targeting may represent a valuable strategy for enhancing the anti-tumor activity of the anti-angiogenic drugs currently used in the clinic for advanced HCC treatment.

Despite the efficacy of both abemaciclib/lenvatinib and ribociclib/lenvatinib combinations, HCC cells, especially SNU398 cells, were relatively less sensitive to ribociclib than abemaciclib, confirming literature data showing that abemaciclib is the most potent CDK4/6 inhibitor (19). Therefore, in this study we mainly focused on the effects of abemaciclib in combination with lenvatinib.

Comparing different schedules of treatment, we demonstrated that the best strategy for combining abemaciclib with lenvatinib was represented by the simultaneous treatment, whereas the sequential schedules (abemaciclib before or after lenvatinib, or pre-treatment with single drugs followed by the combination) in some cases were even less effective than single-agent treatments. It is worth noting that, in contrast with palbociclib, whose current clinical regimen requires a week of drug holiday, the lower toxicity of abemaciclib allows for continuous dosing (39), suggesting that combined treatment with abemaciclib and lenvatinib is potentially feasible in the clinic. In this context, it is of interest to note that a phase II clinical trial is currently ongoing to evaluate the combination of abemaciclib with nivolumab, an ICI directed against PD-1, in HCC patients (NCT03781960).

Mechanistically, the effectiveness of abemaciclib/lenvatinib combination was associated with a stronger dephosphorylation/activation of Rb in comparison with single treatments, which resulted in a significant downregulation of c-myc protein, a known transcriptional target of E2F (34). This effect was observed in all cell models analyzed. In contrast, inhibition of other survival/proliferation signaling pathways contributed to the anti-cancer activity of the drug combination in a cell type-dependent fashion. Indeed, only HUH7 and SNU398 cells showed a marked downregulation of ERK1/2 signaling. In a recent study, lenvatinib was shown to activate feedback signaling through EGFR-PAK2-ERK1/2 and EGFR-PAK2-ERK5 pathways in EGFR expressing HCC cell lines (40). Combining lenvatinib with EGFR inhibitors prevented the activation of this feedback mechanism, ensuring a more complete inhibition of MEK and ERK phosphorylation, which resulted in potentiated anti-proliferative effects both *in vitro* and *in vivo*. The src/FAK signaling was downregulated by abemaciclib and lenvatinib combination only in SNU398 and HepG2 cells. Interestingly, previous studies indicate that FAK represents a valuable druggable target in HCC, being frequently overexpressed in this type of cancer (41).

A variety of evidence indicates that inhibition of CDK4/6, after arresting the cell cycle in the G1 phase, can induce quiescence, senescence, or apoptosis, depending on the cell context, cell type, and duration of the inhibition (42–44).

Here we show that abemaciclib and lenvatinib combination in HUH7 cells promoted senescence in a significant proportion of the cell population (~70%), as demonstrated by the induction of SA-β-gal activity. The molecular mechanism underlying this phenomenon involves the induction of p21, whose role in the positive regulation of senescence is well known (45). Abemaciclib/lenvatinib combination may promote the induction of p21 through two possible mechanisms: downregulation of c-myc protein, which inhibits p21 transcription through direct binding to its promoter (46), and downregulation of MDM2, which acts as a negative regulator of p21 favoring its degradation independently of both p53 and ubiquitination (32). Interestingly, a study by Kovatcheva et al. (47) demonstrated that MDM2 degradation was required for CDK4/6 inhibition to mediate the transition from quiescence to senescence in multiple cell models, including liposarcoma, breast, glioma, and lung cancer cell lines. CDK4/6 inhibition enhanced MDM2 proteasome-dependent turnover through a p53-independent mechanism requiring E3 ligase activity of MDM2 and ATRX expression. It is important to stress that these regulatory processes occur independently of p53, since HUH7 cells are p53 mutant (31). The combination of abemaciclib with lenvatinib significantly reduced the phosphorylation of MDM2 at Ser166. Phosphorylation at this site, required for MDM2 stabilization and inhibition of its self-ubiquitination, is generally attributed to AKT (48); however, in normal liver and HCC cells it was shown to be mediated by MEK-ERK signaling (49). Therefore, downregulation of the MAPK pathway by abemaciclib/lenvatinib combination may contribute to the reduced phosphorylated and total MDM2 levels observed in HUH7 cells.

HUH7 abemaciclib/lenvatinib-treated cells showed a significant increase in ROS levels in comparison with single-agent treatments. ROS have an established role in senescence and their increased production has been shown to be mediated by p21 independently of p53 (50, 51). Therefore, in our experimental system, p21 may induce senescence through a mechanism involving ROS accumulation. It is worth noting that lenvatinib alone induced p21 accumulation, confirming previous findings in thyroid cancer (52), and promoted ROS production, without inducing senescence. ROS were previously shown to play a role in lenvatinib-mediated anti-tumor effects in HUH7 cells, and their accumulation by lenvatinib, as well as by sorafenib and regorafenib, was associated with Keap1-mediated downregulation of Nrf2, a transcription factor playing a key role in the control of antioxidant responses (53).

Growing evidence indicates that induction of senescence exerts anti-tumor effects in HCC (54). For example, inhibition of Sirtuin 6 reduced the tumorigenicity of HCC cells by inducing cellular senescence *via* upregulation of p21 (55). In another study, dual-specificity phosphatase 16 (DUSP16) was shown to play a role in the growth of HCC cells by inactivating p53 and Rb and promoting cell escape from senescence. Accordingly, DUSP16 expression levels were found upregulated in liver cancer and positively correlated with the tumor cell proliferation index (56). It is worth underlining that the aberrant persistence of senescent cells may have detrimental effects with a negative impact on long-term patient outcome. However, induction of senescence can be exploited *in vivo* as a tool for preparing the conditions for subsequent elimination of cancer cells by recruited immune cells (57). In this regard, it is interesting to note that a mechanism contributing to the anti-tumor activity of lenvatinib involves the enhancement of tumor infiltration by NK cells (58). Therefore, we can speculate that, in the therapeutic strategy proposed in our study, lenvatinib may favor the elimination of senescent cells induced by abemaciclib/lenvatinib combination by stimulating the recruitment of tumor-infiltrating NK cells *in vivo*; this may help avoid senescence-related side effects and minimize the risk of regression.

## Data Availability

The raw data supporting the conclusions of this article will be made available by the authors, without undue reservation.

## References

[1] Hatanaka T Naganuma A Kakizaki S. Lenvatinib for hepatocellular carcinoma: A literature review. Pharm (Basel) (2021) 14(1). doi: 10.3390/ph14010036 PMC7825021 33418941

[2] Petrick JL Florio AA Znaor A Ruggieri D Laversanne M Alvarez CS. International trends in hepatocellular carcinoma incidence, 1978-2012. Int J Cancer (2020) 147(2):317–30. doi: 10.1002/ijc.32723 PMC7470451 31597196

[3] Villanueva A. Hepatocellular carcinoma. N Engl J Med (2019) 380(15):1450–62. doi: 10.1056/NEJMra1713263 30970190

[4] Huang A Yang XR Chung WY Dennison AR Zhou J. Targeted therapy for hepatocellular carcinoma. Signal Transduct Target Ther (2020) 5(1):146. doi: 10.1038/s41392-020-00264-x 32782275 PMC7419547

[5] Zhang H Zhang W Jiang L Chen Y. Recent advances in systemic therapy for hepatocellular carcinoma. biomark Res (2022) 10(1):3. doi: 10.1186/s40364-021-00350-4 35000616 PMC8744248

[6] Finn RS Qin S Ikeda M Galle PR Ducreux M Kim TY. Atezolizumab plus bevacizumab in unresectable hepatocellular carcinoma. N Engl J Med (2020) 382(20):1894–905. doi: 10.1056/NEJMoa1915745 32402160

[7] Kudo M Ueshima K Yokosuka O Ogasawara S Obi S Izumi N. Sorafenib plus low-dose cisplatin and fluorouracil hepatic arterial infusion chemotherapy versus sorafenib alone in patients with advanced hepatocellular carcinoma (Silius): a randomised, open label, phase 3 trial. Lancet Gastroenterol Hepatol (2018) 3(6):424–32. doi: 10.1016/S2468-1253(18)30078-5 29631810

[8] Zhao Y Zhang YN Wang KT Chen L. Lenvatinib for hepatocellular carcinoma: From preclinical mechanisms to anti-cancer therapy. Biochim Biophys Acta Rev Cancer (2020) 1874(1):188391. doi: 10.1016/j.bbcan.2020.188391 32659252

[9] Kimura T Kato Y Ozawa Y Kodama K Ito J Ichikawa K. Immunomodulatory activity of lenvatinib contributes to antitumor activity in the Hepa1-6 hepatocellular carcinoma model. Cancer Sci (2018) 109(12):3993–4002. doi: 10.1111/cas.13806 30447042 PMC6272102

[10] Finn RS Ikeda M Zhu AX Sung MW Baron AD Kudo M. Phase ib study of lenvatinib plus pembrolizumab in patients with unresectable hepatocellular carcinoma. J Clin Oncol (2020) 38(26):2960–70. doi: 10.1200/JCO.20.00808 PMC7479760 32716739

[11] Llovet JM Kelley RK Villanueva A Singal AG Pikarsky E Roayaie S. Hepatocellular carcinoma. Nat Rev Dis Primers (2021) 7(1):6. doi: 10.1038/s41572-020-00240-3 33479224 PMC13537433

[12] Lu JW Lin YM Chang JG Yeh KT Chen RM Tsai JJ. Clinical implications of deregulated Cdk4 and cyclin D1 expression in patients with human hepatocellular carcinoma. Med Oncol (2013) 30(1):379. doi: 10.1007/s12032-012-0379-5 23292829

[13] Azechi H Nishida N Fukuda Y Nishimura T Minata M Katsuma H. Disruption of the P16/Cyclin D1/Retinoblastoma protein pathway in the majority of human hepatocellular carcinomas. Oncology (2001) 60(4):346–54. doi: 10.1159/000058531 11408803

[14] Zhou Y Wang XB Qiu XP Shuai Z Wang C Zheng F. Cdkn2a promoter methylation and hepatocellular carcinoma risk: A meta-analysis. Clin Res Hepatol Gastroenterol (2018) 42(6):529–41. doi: 10.1016/j.clinre.2017.07.003 30143452

[15] Totoki Y Tatsuno K Covington KR Ueda H Creighton CJ Kato M. Trans-ancestry mutational landscape of hepatocellular carcinoma genomes. Nat Genet (2014) 46(12):1267–73. doi: 10.1038/ng.3126 25362482

[16] Sobhani N D'Angelo A Pittacolo M Roviello G Miccoli A Corona SP. Updates on the Cdk4/6 inhibitory strategy and combinations in breast cancer. Cells (2019) 8(4). doi: 10.3390/cells8040321 PMC6523967 30959874

[17] Bollard J Miguela V Ruiz de Galarreta M Venkatesh A Bian CB Roberto MP. Palbociclib (Pd-0332991), a selective Cdk4/6 inhibitor, restricts tumour growth in preclinical models of hepatocellular carcinoma. Gut (2017) 66(7):1286–96. doi: 10.1136/gutjnl-2016-312268 PMC5512174 27849562

[18] Digiacomo G Fumarola C La Monica S Bonelli MA Cretella D Alfieri R. Simultaneous combination of the Cdk4/6 inhibitor palbociclib with regorafenib induces enhanced anti-tumor effects in hepatocarcinoma cell lines. Front Oncol (2020) 10:563249. doi: 10.3389/fonc.2020.563249 33072590 PMC7539564

[19] Braal CL Jongbloed EM Wilting SM Mathijssen RHJ Koolen SLW Jager A. Inhibiting Cdk4/6 in breast cancer with palbociclib, ribociclib, and abemaciclib: Similarities and differences. Drugs (2021) 81(3):317–31. doi: 10.1007/s40265-020-01461-2 PMC7952354 33369721

[20] Cretella D Ravelli A Fumarola C La Monica S Digiacomo G Cavazzoni A. The anti-tumor efficacy of Cdk4/6 inhibition is enhanced by the combination with Pi3k/Akt/Mtor inhibitors through impairment of glucose metabolism in tnbc cells. J Exp Clin Cancer Res (2018) 37(1):72. doi: 10.1186/s13046-018-0741-3 29587820 PMC5872523

[21] Van Der Steen N Leonetti A Keller K Dekker H Funel N Lardon F. Decrease in phospho-Pras40 plays a role in the synergy between erlotinib and crizotinib in an egfr and cmet wild-type squamous non-small cell lung cancer cell line. Biochem Pharmacol (2019) 166:128–38. doi: 10.1016/j.bcp.2019.05.014 31078602

[22] La Monica S Fumarola C Cretella D Bonelli M Minari R Cavazzoni A. Efficacy of the Cdk4/6 dual inhibitor abemaciclib in egfr-mutated nsclc cell lines with different resistance mechanisms to osimertinib. Cancers (Basel) (2020) 13(1). doi: 10.3390/cancers13010006 PMC7792603 33374971

[23] Cavazzoni A Alfieri RR Carmi C Zuliani V Galetti M Fumarola C. Dual mechanisms of action of the 5-benzylidene-hydantoin upr1024 on lung cancer cell lines. Mol Cancer Ther (2008) 7(2):361–70. doi: 10.1158/1535-7163.MCT-07-0477 18281519

[24] Fumarola C Bozza N Castelli R Ferlenghi F Marseglia G Lodola A. Expanding the arsenal of fgfr inhibitors: a novel chloroacetamide derivative as a new irreversible agent with anti-proliferative activity against fgfr1-amplified lung cancer cell lines. Front Oncol (2019) 9:179. doi: 10.3389/fonc.2019.00179 30972293 PMC6443895

[25] La Monica S Caffarra C Saccani F Galvani E Galetti M Fumarola C. Gefitinib inhibits invasive phenotype and epithelial-mesenchymal transition in drug-resistant nsclc cells with met amplification. PloS One (2013) 8(10):e78656. doi: 10.1371/journal.pone.0078656 24167634 PMC3805532

[26] Bonelli MA Digiacomo G Fumarola C Alfieri R Quaini F Falco A. Combined inhibition of Cdk4/6 and Pi3k/Akt/Mtor pathways induces a synergistic anti-tumor effect in malignant pleural mesothelioma cells. Neoplasia (2017) 19(8):637–48. doi: 10.1016/j.neo.2017.05.003 PMC5508477 28704762

[27] Finn RS Dering J Conklin D Kalous O Cohen DJ Desai AJ. Pd 0332991, a selective cyclin d kinase 4/6 inhibitor, preferentially inhibits proliferation of luminal estrogen receptor-positive human breast cancer cell lines in vitro. Breast Cancer Res (2009) 11(5):R77. doi: 10.1186/bcr2419 19874578 PMC2790859

[28] Pan Q Sathe A Black PC Goebell PJ Kamat AM Schmitz-Draeger B. Cdk4/6 inhibitors in cancer therapy: A novel treatement strategy for bladder cancer. Bladder Cancer (2017) 3(2):79–88. doi: 10.3233/BLC-170105 28516152 PMC5409046

[29] Matsuki M Hoshi T Yamamoto Y Ikemori-Kawada M Minoshima Y Funahashi Y. Lenvatinib inhibits angiogenesis and tumor fibroblast growth factor signaling pathways in human hepatocellular carcinoma models. Cancer Med (2018) 7(6):2641–53. doi: 10.1002/cam4.1517 PMC6010799 29733511

[30] Kumari R Jat P. Mechanisms of cellular senescence: Cell cycle arrest and senescence associated secretory phenotype. Front Cell Dev Biol (2021) 9:645593. doi: 10.3389/fcell.2021.645593 33855023 PMC8039141

[31] Hsu IC Tokiwa T Bennett W Metcalf RA Welsh JA Sun T. P53 gene mutation and integrated hepatitis b viral DNA sequences in human liver cancer cell lines. Carcinogenesis (1993) 14(5):987–92. doi: 10.1093/carcin/14.5.987 8389256

[32] Zhang Z Wang H Li M Agrawal S Chen X Zhang R. Mdm2 is a negative regulator of P21waf1/Cip1, independent of P53. J Biol Chem (2004) 279(16):16000–6. doi: 10.1074/jbc.M312264200 14761977

[33] Lu T Finkel T. Free radicals and senescence. Exp Cell Res (2008) 314(9):1918–22. doi: 10.1016/j.yexcr.2008.01.011 PMC2486428 18282568

[34] Oswald F Lovec H Moroy T Lipp M. E2f-dependent regulation of human myc: trans-activation by cyclins d1 and a overrides tumour suppressor protein functions. Oncogene (1994) 9(7):2029–36. 8208548

[35] Bahrami BF Ataie-Kachoie P Pourgholami MH Morris DL. P70 ribosomal protein S6 kinase (Rps6kb1): An update. J Clin Pathol (2014) 67(12):1019–25. doi: 10.1136/jclinpath-2014-202560 25100792

[36] Goel S Wang Q Watt AC Tolaney SM Dillon DA Li W. Overcoming therapeutic resistance in her2-positive breast cancers with cdk4/6 inhibitors. Cancer Cell (2016) 29(3):255–69. doi: 10.1016/j.ccell.2016.02.006 PMC4794996 26977878

[37] Westhoff MA Serrels B Fincham VJ Frame MC Carragher NO. Src-mediated phosphorylation of focal adhesion kinase couples actin and adhesion dynamics to survival signaling. Mol Cell Biol (2004) 24(18):8113–33. doi: 10.1128/MCB.24.18.8113-8133.2004 PMC515031 15340073

[38] Nenni M Oncul S Ercan A Celebier M Suslu I Haznedaroglu IC. Exposure of hepatocellular carcinoma cells to ankaferd blood stopper® alters cell death signaling networks confirmed by oncoproteomic and genomic profiling studies. Curr Traditional Med (2021) 7(2):8. doi: 10.2174/2215083806666200117093815

[39] Onesti CE Jerusalem G. Cdk4/6 inhibitors in breast cancer: differences in toxicity profiles and impact on agent choice. a systematic review and meta-analysis. Expert Rev Anticancer Ther (2021) 21(3):283–98. doi: 10.1080/14737140.2021.1852934 33233970

[40] Jin H Shi Y Lv Y Yuan S Ramirez CFA Lieftink C. Egfr activation limits the response of liver cancer to lenvatinib. Nature (2021) 595(7869):730–4. doi: 10.1038/s41586-021-03741-7 34290403

[41] Romito I Porru M Braghini MR Pompili L Panera N Crudele A. Focal adhesion kinase inhibitor tae226 combined with sorafenib slows down hepatocellular carcinoma by multiple epigenetic effects. J Exp Clin Cancer Res (2021) 40(1):364. doi: 10.1186/s13046-021-02154-8 34784956 PMC8597092

[42] Klein ME Kovatcheva M Davis LE Tap WD Koff A. Cdk4/6 inhibitors: The mechanism of action may not be as simple as once thought. Cancer Cell (2018) 34(1):9–20. doi: 10.1016/j.ccell.2018.03.023 29731395 PMC6039233

[43] Blagosklonny MV. Geroconversion: irreversible step to cellular senescence. Cell Cycle (2014) 13(23):3628–35. doi: 10.4161/15384101.2014.985507 PMC4614001 25483060

[44] Yoshida A Diehl JA. Cdk4/6 inhibitor: From quiescence to senescence. Oncoscience (2015) 2(11):896–7. doi: 10.18632/oncoscience.256 PMC4675777 26697514

[45] Bonelli M La Monica S Fumarola C Alfieri R. Multiple effects of cdk4/6 inhibition in cancer: from cell cycle arrest to immunomodulation. Biochem Pharmacol (2019) 170:113676. doi: 10.1016/j.bcp.2019.113676 31647925

[46] Gartel AL Ye X Goufman E Shianov P Hay N Najmabadi F. Myc represses the P21(Waf1/Cip1) promoter and interacts with Sp1/Sp3. Proc Natl Acad Sci U.S.A. (2001) 98(8):4510–5. doi: 10.1073/pnas.081074898 PMC31865 11274368

[47] Kovatcheva M Liu DD Dickson MA Klein ME O'Connor R Wilder FO. Mdm2 turnover and expression of atrx determine the choice between quiescence and senescence in response to cdk4 inhibition. Oncotarget (2015) 6(10):8226–43. doi: 10.18632/oncotarget.3364 PMC4480747 25803170

[48] Feng J Tamaskovic R Yang Z Brazil DP Merlo A Hess D. Stabilization of Mdm2 *Via* decreased ubiquitination is mediated by protein kinase B/Akt-dependent phosphorylation. J Biol Chem (2004) 279(34):35510–7. doi: 10.1074/jbc.M404936200 15169778

[49] Malmlof M Roudier E Hogberg J Stenius U. Mek-Erk-Mediated phosphorylation of Mdm2 at ser-166 in hepatocytes. Mdm2 Is Activated Response to Inhibited Akt Signaling J Biol Chem (2007) 282(4):2288–96. doi: 10.1074/jbc.M604953200 17107963

[50] Macip S Igarashi M Fang L Chen A Pan ZQ Lee SW. Inhibition of P21-mediated ros accumulation can rescue P21-induced senescence. EMBO J (2002) 21(9):2180–8. doi: 10.1093/emboj/21.9.2180 PMC125979 11980715

[51] Masgras I Carrera S de Verdier PJ Brennan P Majid A Makhtar W. Reactive oxygen species and mitochondrial sensitivity to oxidative stress determine induction of cancer cell death by P21. J Biol Chem (2012) 287(13):9845–54. doi: 10.1074/jbc.M111.250357 PMC3322987 22311974

[52] Kim SY Kim SM Chang HJ Kim BW Lee YS Park CS. Solat (Sorafenib lenvatinib alternating treatment): A new treatment protocol with alternating sorafenib and lenvatinib for refractory thyroid cancer. BMC Cancer (2018) 18(1):956. doi: 10.1186/s12885-018-4854-z 30286728 PMC6172752

[53] Zheng A Chevalier N Calderoni M Dubuis G Dormond O Ziros PG. Crispr/Cas9 genome-wide screening identifies keap1 as a sorafenib, lenvatinib, and regorafenib sensitivity gene in hepatocellular carcinoma. Oncotarget (2019) 10(66):7058–70. doi: 10.18632/oncotarget.27361 PMC6925031 31903165

[54] Liu P Tang Q Chen M Chen W Lu Y Liu Z. Hepatocellular senescence: Immunosurveillance and future senescence-induced therapy in hepatocellular carcinoma. Front Oncol (2020) 10:589908. doi: 10.3389/fonc.2020.589908 33330071 PMC7732623

[55] Feng XX Luo J Liu M Yan W Zhou ZZ Xia YJ. Sirtuin 6 promotes transforming growth factor-beta1/h2o2/hocl-mediated enhancement of hepatocellular carcinoma cell tumorigenicity by suppressing cellular senescence. Cancer Sci (2015) 106(5):559–66. doi: 10.1111/cas.12632 PMC4452156 25683165

[56] Zhang H Zheng H Mu W He Z Yang B Ji Y. Dusp16 ablation arrests the cell cycle and induces cellular senescence. FEBS J (2015) 282(23):4580–94. doi: 10.1111/febs.13518 26381291

[57] Wang C Vegna S Jin H Benedict B Lieftink C Ramirez C. Inducing and exploiting vulnerabilities for the treatment of liver cancer. Nature (2019) 574(7777):268–72. doi: 10.1038/s41586-019-1607-3 PMC6858884 31578521

[58] Zhang Q Liu H Wang H Lu M Miao Y Ding J. Lenvatinib promotes antitumor immunity by enhancing the tumor infiltration and activation of nk cells. Am J Cancer Res (2019) 9(7):1382–95. PMC6682710 31392076

