# Corrigendum: CDK4/6 inhibitors improve the anti-tumor efficacy of lenvatinib in hepatocarcinoma cells

**DOI:** 10.3389/fonc.2024.1532291

**Published:** 2024-12-06

**Authors:** Graziana Digiacomo, Claudia Fumarola, Silvia La Monica, Mara Bonelli, Andrea Cavazzoni, Maricla Galetti, Rita Terenziani, Kamal Eltayeb, Francesco Volta, Silvia Zoppi, Patrizia Bertolini, Gabriele Missale, Roberta Alfieri, Pier Giorgio Petronini

**Affiliations:** ^1^ Department of Medicine and Surgery, University of Parma, Parma, Italy; ^2^ Department of Occupational and Environmental Medicine, Epidemiology and Hygiene, INAIL - Italian Workers’ Compensation Authority, Rome, Italy; ^3^ Paediatric Hematology Oncology Unit, University Hospital of Parma, Parma, Italy; ^4^ Unit of Infectious Diseases and Hepatology, University Hospital of Parma, Parma, Italy

**Keywords:** hepatocarcinoma (HCC), CDK4/6 inhibition, abemaciclib, lenvatinib, senescence

In the published article, there was an error in **Figures 2B** and **3D** as published. Two representative images of colony formation were unintentionally inserted twice during the figure assembly for the manuscript preparation. The images of HepG2 cells shown in **Figure 3D** were inserted by mistake in **Figure 2B** (panel HepG2 cells). The images of HUH7 shown in **Figure 2B** were inserted by mistake in **Figure 3D** (panel SNU398 cells). The corrected **Figure 2B** and **Figure 3D** and their caption appear below.

**Figure 2:**
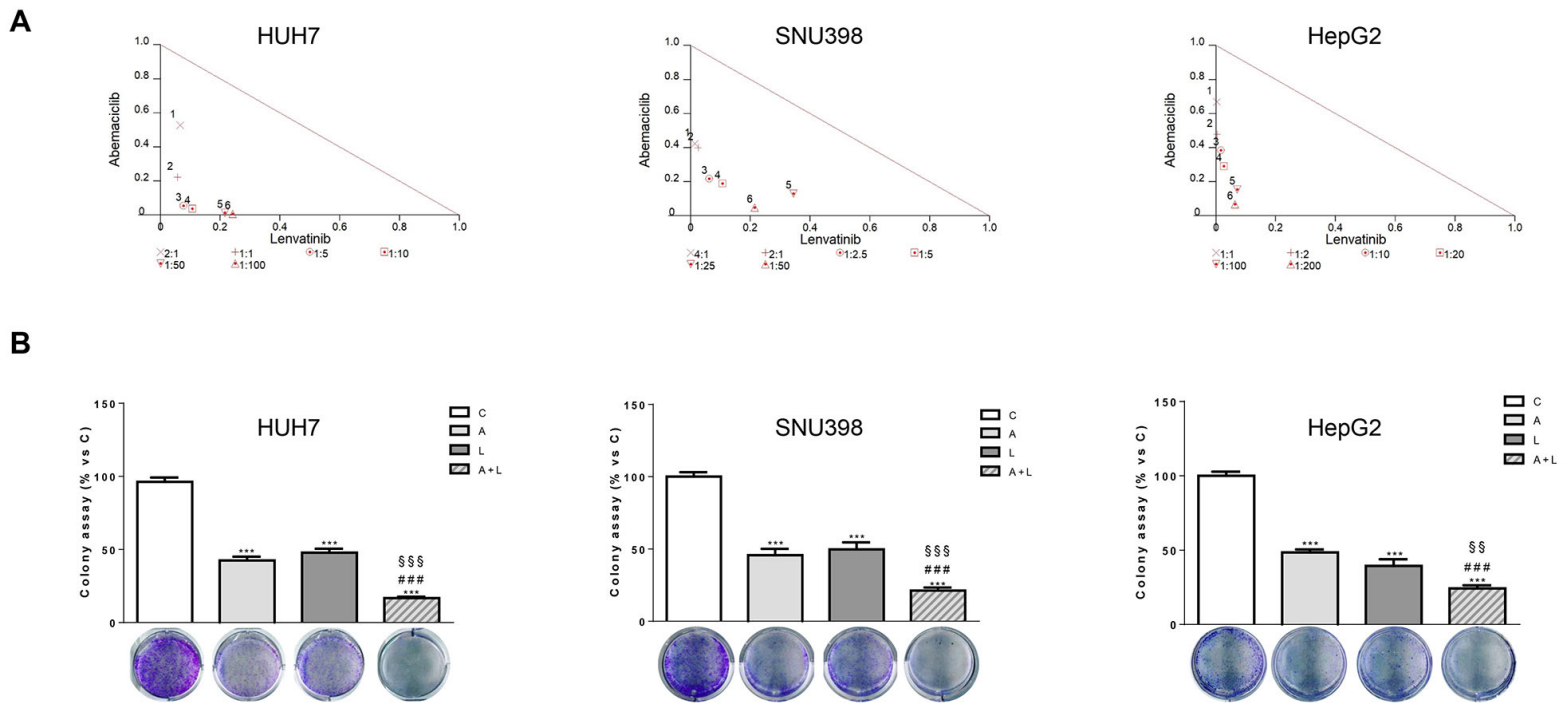
Abemaciclib and lenvatinib combination exerts additive anti-proliferative effects in HCC cells and inhibits colony formation more strongly than single agents. **(A)** Cells were treated with A, L or the combination. The growth medium with drugs was refreshed every 3 days. After 6 days, cell proliferation was assessed by CV assay. Combination indexes were calculated with Calcusyn software. **(B)** HUH7, SNU398, and HepG2 cells were treated with A or L at their corresponding IC_50_ values alone or in combination. After 6 days, colony formation was assessed by CV assay. Representative images of crystal violet staining of colonies are shown. ***p<0.001 vs C, ^###^p<0.001 vs A; ^§§^p< 0.01, ^§§§^p<0.001 vs L. Data in A are representative of three independent experiments. Data in B are mean values ± SD of three independent experiments.

**Figure 3:**
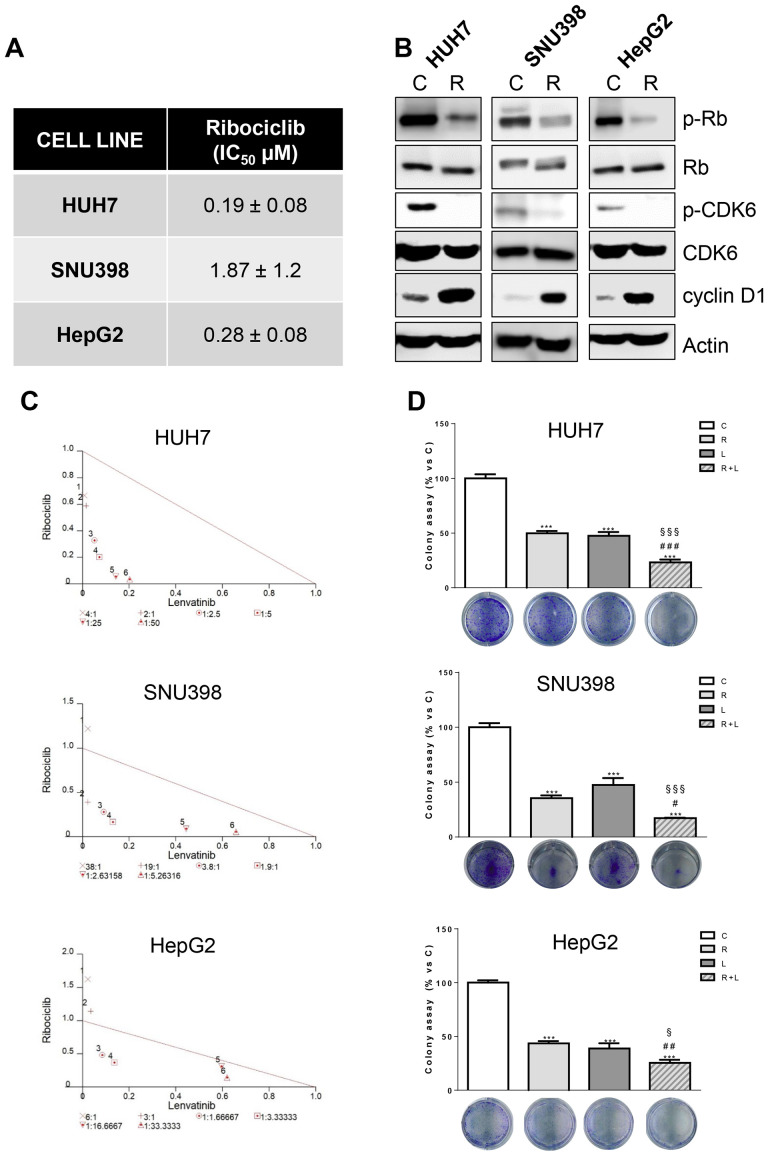
Ribociclib and lenvatinib combination exerts additive anti-proliferative effects in HCC cells and inhibits colony formation more strongly than single agents. **(A)** After 24h from seeding, HUH7, SNU398, and HepG2 cells were treated with increasing concentrations of ribociclib (R) for 6 days. Cells proliferation was evaluated by CV assay and the IC_50_ values were calculated using GraphPad Prism 6.00 software. **(B)** HCC cells were untreated (C) or treated with 1 μM R for 24h. The cells were lysed and the expression of the indicated proteins was evaluated by Western blot analysis. **(C)** Cells were treated with R, L or the combination. The growth medium with drugs was refreshed every 3 days. After 6 days, cell proliferation was assessed by CV assay. Combination indexes were calculated with Calcusyn software. **(D)** HUH7, SNU398, and HepG2 cells were treated with R or L at their corresponding IC_50_ values alone or in combination. After 6 days, colony formation was assessed by CV assay. Representative images of crystal violet staining of colonies are shown. ***p<0.001 vs C; ^#^p<0.05, ^##^p<0.01 ^###^p<0.001 vs R; ^§^p< 0.05, ^§§§^p<0.001 vs L. Data in A are mean values ± SD of three independent experiments. Data in B-C are representative of two independent experiments. Data in D are mean values ± SD of two independent experiments.

The authors apologize for these errors and state that this does not change the scientific conclusions of the article in any way. The original article has been updated.

