# Supplementary material for: CDK4/6 inhibitors improve the anti-tumor efficacy of lenvatinib in hepatocarcinoma cells
Source: Front Oncol. 2022 Jul 22;12:942341. doi: 10.3389/fonc.2022.942341 (PMC9354684; doi:10.3389/fonc.2022.942341)
Supplement: Supplementary file 1 [file DataSheet1.docx]

Supplementary Material


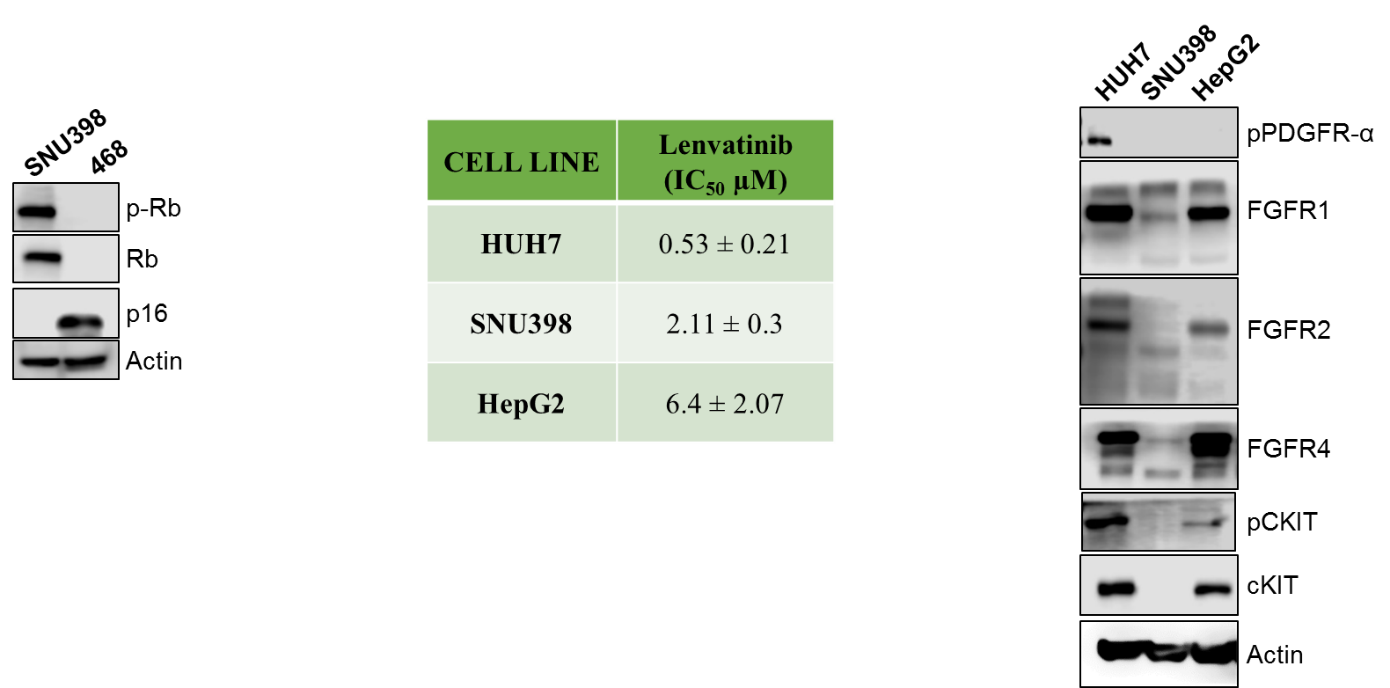
 A B C

**Figure S1.** (A) After 24h from seeding, cells were lysed and the expression of the indicated proteins was evaluated by Western blot analysis. (B) After 24h from seeding, HUH7, SNU398, and HepG2 cells lines were treated with increasing concentrations of lenvatinib (L) for 6 days. Cells proliferation was evaluated by CV assay and the IC50 values were calculated using GraphPad Prism 6.00 software. (C) After 24h from seeding, cells were lysed and the expression of the indicated proteins was evaluated by Western blot analysis. Data in A and C are representative of two independent experiments. Data in B are mean values ±SD of four independent experiments.

A B


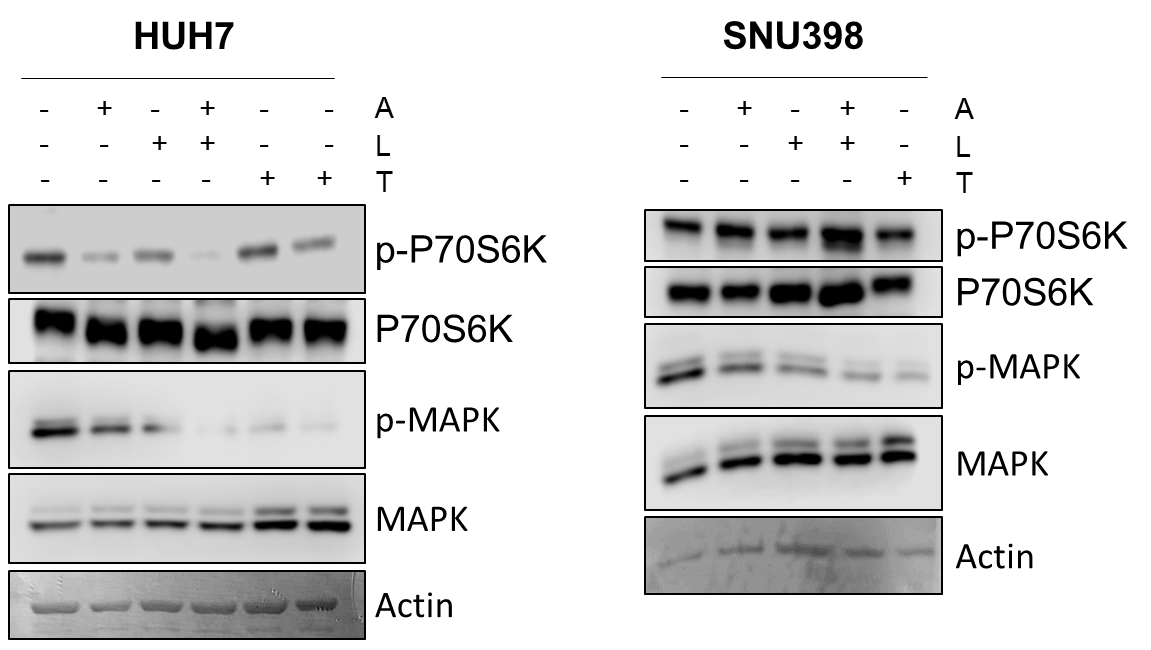


**Figure S2.** HUH7 cells (A) were treated with 1 μM A and 0.5 μM L, alone or in combination, or 50 or 100nM trametinib (T). SNU398 cells (B) were treated with 1 μM A and 2 μM L, alone or in combination, or 100nM trametinib. After 24 h, the cells were lysed and the expression of the indicated proteins was evaluated by Western blot analysis. Data are representative of two independent experiments.
